# Effectiveness of Structured Skin Care Using Silicone Barrier Spray Compared with Zinc Oxide Ointment for Incontinence-Associated Dermatitis in Long-Term Care Residents: A Randomized Controlled Trial

**DOI:** 10.7150/ijms.132180

**Published:** 2026-08-24

**Authors:** Po-Chung Chen, Chi-Ming Chu, Po-Jen Hsiao, Hsieh-Chih Tsai

**Affiliations:** 1 Graduate Institute of Applied Science and Technology, National Taiwan University of Science and Technology, Taipei, Taiwan.; 2 Division of Biostatistics and Medical Informatics, Department of Epidemiology, College of Public Health, National Defense Medical University, Taipei, Taiwan.; 3 Graduate Institute of Medical Sciences, National Defense Medical University, Taipei, Taiwan.; 4 Division of Nephrology, Department of Internal Medicine, Taoyuan Armed Forces General Hospital, Taoyuan, Taiwan.; 5 Division of Nephrology, Department of Internal Medicine, Tri-Service General Hospital, National Defense Medical University, Taipei, Taiwan.; 6 Department of Life Sciences, National Central University, Taoyuan, Taiwan.

**Keywords:** incontinence-associated dermatitis, silicone spray, skin barrier, nursing care, long-term care

## Abstract

**Background:**

Incontinence-associated dermatitis (IAD) is a common and clinically significant condition among the residents of long-term care facilities. Although structured skin care is widely recommended for IAD prevention and management, evidence comparing sprayable silicone barrier films with traditional zinc oxide-based barriers remains limited.

**Methods:**

We conducted a 14-day prospective, randomized controlled trial in a long-term care facility. Forty residents with mild IAD (GLOBIAD Category 1) were randomly assigned in a 1:1 ratio to receive either a silicone-based barrier spray (n = 20) or zinc oxide ointment (n = 20), in addition to standardized cleansing care. IAD severity was assessed using the modified Ghent Global IAD Monitoring Tool (GLOBIAD-M). Secondary outcomes included skin hydration, transepidermal water loss (TEWL), skin pH, and skin temperature, measured at baseline, Day 7, and Day 14. Longitudinal changes were analyzed using generalized estimating equations.

**Results:**

The severity of IAD significantly improved over time in both groups (p<0.001), with no significant between-group differences or group-by-time interaction. A modest increase in skin pH toward a more neutral range was observed over time (p=0.036). Although skin hydration, TEWL, and skin temperature did not show significant changes, their overall patterns remained compatible with the clinical improvement observed during follow-up. No adverse events were observed in either group.

**Conclusion:**

Within the 14-day study period, sprayable silicone barrier film was well tolerated, with no statistically significant differences in clinical outcomes compared with zinc oxide ointment. The ease of application and the absence of mechanical removal may facilitate routine skin care, making sprayable silicone barrier film a suitable option in long-term care settings.

## Introduction

Incontinence-associated dermatitis (IAD) is a type of moisture-associated inflammatory skin condition caused by prolonged exposure to urine or feces 1,2. It affects approximately 20-50% of residents in long-term care facilities, particularly those with limited mobility, cognitive impairment, or multiple comorbidities. Common symptoms include pain, burning, and pruritus. IAD may also increase risk of secondary skin complications, including pressure injuries 2,3.

The development of IAD involves prolonged moisture exposure, chemical irritation, friction, and disruption of the epidermal barrier 3,4. Excess moisture increases skin permeability and makes the affected area more easily damaged by friction. Exposure to urine and feces also increases skin pH and enhances the activity of fecal enzymes, further weakening the barrier. These changes promote microbial colonization and local inflammation, eventually leading to tissue injury. Experimental studies of dermatitis have also identified inflammatory pathways and oxidative stress as contributors to skin damage. In particular, reducing IL-17/CCL2 signaling and oxidative stress may help lessen dermatitis-related skin injury 5.

Current clinical guidelines recommend structured skin care, including gentle cleansing with pH-appropriate products, regular moisturization, and barrier protectant application 6-9. Zinc oxide ointments are commonly used because they form an occlusive protective layer. Their thick consistency, however, can make application and removal difficult, especially in frail older adults, and repeated cleansing may increase friction 10,11. Silicone-based formulations provide a different type of protection. They form a thin, breathable, and water-repellent film that may reduce transepidermal water loss (TEWL) and friction. Sprayable formulations can also be applied without rubbing the affected skin and do not require mechanical removal during routine care 12-15.

Most evidence on silicone-based products for IAD comes from studies of barrier creams, impregnated washcloths, and structured skin care programs 15-17. These interventions may help prevent or treat IAD, but the supporting evidence remains limited. Sprayable products can be applied quickly and evenly without touching or rubbing the affected skin. This feature may be useful when caring for sensitive or difficult-to-reach areas.

Previous clinical studies of silicone-based products for IAD have focused on creams, wipes, and other barrier products rather than sprayable films 18,19. How a topical product is applied may affect treatment outcomes and routine care. A recent study examined radiofrequency-assisted ointment delivery for chronic dermatitis and showed the potential importance of delivery methods in topical treatment 20. Although that study examined a different treatment and skin condition, it suggests that the method of application may be important. Silicone sprays can cover the affected area without direct rubbing. This may reduce friction and facilitate skin care for long-term care residents with fragile skin. Therefore, this randomized controlled trial aimed to evaluate whether the adjunctive use of a sprayable silicone barrier film improves clinical and biophysical outcomes compared to standard zinc oxide-based care in residents with mild IAD in a long-term care setting.

## Methods

### Participants and randomization

Trial Registration: This study was registered retrospectively at ClinicalTrials.gov on June 9, 2026 (NCT07637695), as the participant enrollment had been completed prior to registration. However, the study protocol, eligibility criteria, interventions, and outcome measures remained strictly unchanged throughout the study period. This prospective RCT was conducted in a long-term care facility in northern Taiwan between January 1, 2023, and November 30, 2023. Eligible participants were long-term care residents diagnosed with mild IAD, classified as GLOBIAD Category 1. Residents with Category 2 IAD (skin loss or erosion) were excluded 21. After obtaining written informed consent, participants were randomly assigned in a 1:1 ratio to the experimental or control group using a computer-generated random sequence. The allocation was concealed using sealed opaque envelopes prepared by an independent investigator who was not involved in participant enrollment or outcome assessment. Owing to the distinct physical formulations of the interventions (spray versus ointment), blinding of the bedside caregivers was not feasible. However, to minimize potential assessment and observer biases, all clinical evaluations and biophysical measurements were performed by a designated outcome assessor who was blinded to the group allocation throughout the 14-day study period.

### Interventions

All participants received a standardized structured skin care regimen. Following each episode of incontinence or a change in the absorbent product, the perineal skin was gently cleansed with wet towels and a pH-balanced moisturizing cleanser. Subsequently, barrier protection was administered according to group allocation:

**Experimental Group:** A silicone-based barrier film spray (Brava® Skin Barrier Spray, Coloplast A/S, Denmark) was applied after each cleansing.

**Control Group:** Traditional barrier protection using zinc oxide ointment was applied after each cleansing.

### Outcome measures

**Clinical Assessment:** Skin condition was assessed on Days 1, 7, and 14 using the modified GLOBIAD-M tool, with scores of 0 (normal), 1 (Category 1A), or 2 (Category 1B) 21.

**Biophysical Parameters:** Biophysical measures included TEWL, skin hydration, skin pH and skin temperature. All measurements were performed on Days 1, 7, and 14. TEWL was measured using a closed-chamber evaporimeter, and skin hydration was assessed using stratum corneum capacitance. The skin pH and temperature were measured using a flat-surface electrode device. Measurements were performed in triplicate at the affected sites, and the mean values were used for the analysis. To ensure data consistency, all participants remained in a climate-controlled room (ambient temperature: 22-24°C; relative humidity: 40-60%) for at least 15 minutes prior to assessment.

### Statistical analysis

As no previous randomized controlled trials have specifically evaluated this sprayable silicone barrier film for mild IAD in long-term care settings, this study was designed as a pilot trial. Therefore, a convenience sample of 40 participants was enrolled to generate preliminary estimates of treatment effects and clinical outcomes. No formal a priori sample size calculation was performed, and the effect estimates and corresponding 95% confidence intervals derived from the GEE models were intended to inform sample size planning for future adequately powered multicenter trials.

Statistical analyses were performed using SPSS version 26.0. Between-group differences at baseline were assessed using Student's t-test, the chi-square test, or Fisher's exact test, as appropriate. Longitudinal changes were analyzed using generalized estimating equations (GEE). Regression coefficients (β), corresponding 95% confidence intervals (95% CIs), and ***p*****-**values were reported **where applicable**. Statistical significance was defined as p<0.05.

## Results

### Participant flow and baseline characteristics

The participant flow diagram is presented in Figure 1. Of the 52 residents assessed for eligibility, 40 met the inclusion criteria and underwent randomization, with 20 assigned to each treatment group. One participant in the experimental group died before the Day 7 assessment for reasons unrelated to the study intervention. Consequently, 19 participants in the experimental group completed the study protocol. In contrast, all 20 participants in the control group successfully completed the 14-day follow-up. In accordance with the intent-to-treat (ITT) principle, statistical analysis via generalized estimating equations (GEE) was performed using the baseline and available follow-up data from all 40 randomized participants (n = 20 per group). The mean age of participants was 81.17 ± 10.46 years, and 70% of the patients were female. Demographic and clinical characteristics, including comorbidities, such as cardiovascular disease, diabetes, dementia, and stroke, were similar between the groups (all p > 0.05) (Table 1).

### Baseline biophysical parameters

At baseline, no significant differences were observed between the two groups (Table 2). Mean hydration was 50.99 ± 36.35 in the experimental group and 51.66 ± 38.44 in the control group (p = 0.955). TEWL was also comparable (65.21 ± 76.21 vs. 71.78 ± 112.09 g/m²/h, p = 0.830). Skin pH showed no significant difference between groups (5.76 ± 0.84 vs. 5.46 ± 0.82, p = 0.264). Skin temperature was similar (31.09 ± 1.26 °C vs. 30.97 ± 1.45 °C, p = 0.791). IAD severity score was identical at baseline (1.00 ± 0.00 in both groups).

### IAD severity

Mean IAD severity scores remained stable at 1.00 at Day 1 and Day 7, but declined significantly to 0.62 ± 0.54 at Day 14 (Table 3, Figure 2). The time effect was highly significant (p < 0.001), indicating a progressive improvement across the study period. In contrast, neither the between-group difference at Day 14 (p = 0.855) nor the group × time interaction (p = 0.855) was statistically significant. Both groups showed similar patterns of clinical improvement over time. As shown in Figure 2, both treatment groups followed a similar downward trend in IAD severity by Day 14.

**Figure 3 shows representative clinical photographs from a participant in the experimental group. Compared with the baseline assessment (GLOBIAD Category 1A), the affected skin showed marked improvement by Day 14.**

### Hydration

Hydration levels fluctuated slightly throughout the study, with values of 51.32 ± 36.93 on Day 1, 46.33 ± 35.28 on Day 7, and 47.05 ± 39.11 on Day 14. Hydration did not change significantly over time (p = 0.734). Neither the between-group difference at Day 14 (p = 0.898) nor the group × time interaction (p = 0.985) was significant (Table 3, Figure 4). Hydration decreased slightly from baseline to Day 7 and then increased modestly by Day 14. Both groups followed a similar pattern.

### TEWL

TEWL values decreased from 68.49 ± 94.67 g/m²/h on Day 1 to 37.97 ± 31.65 g/m²/h on Day 14 (Table 3, Figure 5). TEWL decreased in both groups from baseline to Day 7 and changed little thereafter (Figure 5). The numerical decrease was greater in the experimental group, but both groups showed similar changes over time. GEE analysis showed no significant time effect (p = 0.320), between-group difference at Day 14 (p = 0.798), or group × time interaction (p = 0.885).

### Skin pH

Skin pH increased over time in both groups (Figure 6). The overall mean increased from 5.61 ± 0.83 at baseline to 6.00 ± 0.81 on Day 14. GEE analysis showed a significant time effect (p = 0.036). Neither the between-group difference at Day 14 (p = 0.801) nor the group × time interaction (p = 0.355) was statistically significant.

### Skin temperature

Skin temperature exhibited minor fluctuations throughout the study, increasing from 31.03 ± 1.34 °C at Day 1 to 32.13 ± 3.14 °C at Day 14 (Table 3, Figure 7). However, skin temperature did not change significantly over time (p = 0.378). Neither the between-group difference at Day 14 (p = 0.222) nor the group × time interaction (p = 0.150) was statistically significant. A graphical inspection of Figure 7 revealed a small dip in temperature on Day 7, followed by an increase on Day 14. Both groups demonstrated similar fluctuation over time. However, the experimental group exhibited a slightly greater increase in skin temperature by Day 14 than the control group, suggesting a trend toward greater numerical change in skin temperature in the experimental group, although this difference was not statistically significant.

A schematic overview of the structured skin care intervention and key findings is shown in Figure 8.

## Discussion

In this randomized controlled trial, both silicone barrier spray and zinc oxide ointment, when incorporated into a structured skin care regimen, were associated with significant clinical improvement in mild incontinence-associated dermatitis over a 14-day period. The absence of significant between-group differences suggests that structured skin care itself plays a central role in the resolution of early stage IAD, which is consistent with the current guideline recommendations that emphasize cleansing, moisture control, and barrier protection. Several factors may explain the lack of detectable superiority of silicone barrier spray. First, all enrolled participants had mild disease (GLOBIAD Category 1), a stage that typically responds well to appropriate skin care interventions, thereby creating a potential ceiling effect. Because mild IAD often resolves with consistent structured cleansing and basic skin protection alone, detecting product-specific statistically significant differences between the two active barrier agents was inherently difficult in this patient cohort. Second, the relatively short follow-up period was sufficient to capture early clinical improvement but may have been inadequate to detect differences in recurrence prevention, sustained skin barrier recovery, long-term tolerability, or practical outcomes such as a reduction in nursing workload and improved ease of care over an extended duration. Third, although the modified GLOBIAD-M scale is practical for longitudinal assessment, it may lack the sensitivity for detecting subtle differences in erythema severity or skin integrity between treatment modalities. Our results are consistent with previous studies of IAD management. Both Beeckman *et al*. 6 and Kon *et al*. 16 reported significant reductions in IAD severity with structured skin care, regardless of the barrier product used. These studies support regular cleansing and barrier protection as basic components of IAD care. Earlier studies mainly evaluated dimethicone-impregnated washcloths or barrier creams 6,15. In this study, we evaluated a sprayable silicone barrier film as part of structured skin care for early-stage IAD.

Although no statistically significant between-group differences were observed in the biophysical parameters, the overall trends were consistent with the proposed mechanisms for silicone-based barrier films. The gradual reduction in transepidermal water loss observed in both groups may reflect recovery of epidermal barrier function, as reported in previous studies on IAD and skin barrier repair 22,23. The numerically greater decline in TEWL in the silicone spray group may reflect reduced water vapor diffusion and surface friction. However, this observation remains exploratory and requires confirmation in larger studies. Skin surface pH increased toward neutrality in both groups during the intervention period. While normalization of pH may reflect recovery from acute inflammation, sustained elevations in pH have been associated with impaired barrier function and increased microbial colonization 24-26. Skin pH can provide additional information during IAD assessment because visual findings may not fully reflect skin barrier damage. Zinc oxide ointments are effective barrier products, but their thick consistency can make them difficult to apply and remove, especially in frail older adults. Repeated removal may also increase friction. Silicone barrier sprays form a thin, transparent film and do not need to be rubbed off. This may reduce friction during routine skin care. These differences may be important in long-term care settings, where residents require frequent skin care and staffing resources may be limited 27-29.

The way a topical product is applied may affect the treatment response and routine skin care. A recent study reported improved outcomes with radiofrequency-assisted ointment application in chronic dermatitis 20. This study examined a different treatment and skin condition, but it suggests that the method of application may be important. In our study, the silicone spray was applied without rubbing the affected skin. This may help reduce irritation during routine care. Silicone barrier films also differ physically from petrolatum- and zinc oxide-based ointments 6,9. They form a thin, semi-permeable layer that protects the skin from urine and feces while allowing water vapor to pass through 2,6. This protective layer may help maintain the local skin environment and reduce friction during cleansing or repositioning, especially in older adults with fragile skin 30,31.

Guo *et al*. 32 reported an association between maternal constipation and the risk of atopic dermatitis in offspring. Li *et al*. 5 found that botanical extracts reduced ultraviolet B-induced skin injury in mice through effects on inflammatory pathways and oxidative stress. These studies examined different diseases and did not directly evaluate IAD or barrier products. They provide broader background on the factors involved in inflammatory skin injury.

The present trial focused on routine barrier protection for long-term care residents with IAD. Both treatment groups showed clinical improvement, and no statistically significant difference was detected between silicone barrier spray and zinc oxide ointment. The spray could be applied without rubbing the affected skin, which may be useful during routine care. Inflammatory biomarkers were not measured in this study. Therefore, the findings do not establish an anti-inflammatory mechanism and should be interpreted only in relation to the observed clinical and biophysical outcomes.

This study has some limitations. The relatively small sample size reduced statistical power, particularly for secondary biophysical outcomes, including TEWL, as well as skin hydration, pH, and temperature. For this reason, the absence of a statistically significant difference between the two groups should be considered preliminary and should not be interpreted as evidence of equivalence. Second, this was a single-center study with a follow-up period of only 14 days. This period captured early clinical improvement. However, it was too short to assess recurrence, sustained skin protection, or the durability of treatment effects. The modified GLOBIAD-M scale also had a narrow scoring range of 0, 1, or 2. This may have limited its ability to detect small changes in erythema or gradual skin recovery. Future studies should use a more detailed severity scale and objective digital image analysis. Finally, the caregivers could not be blinded because the two products were visibly different. This may have introduced performance bias. Patient-reported outcomes and health economic outcomes were not evaluated and should be included in future studies.

This prospective randomized trial provides preliminary data on the use of sprayable silicone barrier film within a structured skin care program for mild IAD in long-term care residents. The clinical and biophysical assessments produced effect estimates that may help plan future research. Larger multicenter trials should include more participants and longer follow-up periods. Patient-reported outcomes and health economic measures should also be evaluated 33-38. The effect estimates and 95% CIs from this study may be used to calculate the sample size required for these trials.

## Conclusion

Both groups showed clinical improvement over the 14-day study period after receiving structured skin care with either sprayable silicone barrier film or zinc oxide ointment. Regular cleansing, moisture control, and barrier protection remain important in the management of early IAD. The spray can be applied without rubbing the affected skin and does not require mechanical removal. These features may reduce friction during repeated skin care. Although no statistically significant superiority was demonstrated during the 14-day study period, silicone barrier spray may represent a feasible alternative for IAD management in long-term care settings. Larger multicenter studies with longer follow-up periods are warranted to further evaluate its long-term clinical, biophysical, and practical benefits.

## Figures and Tables

**Figure 1:**
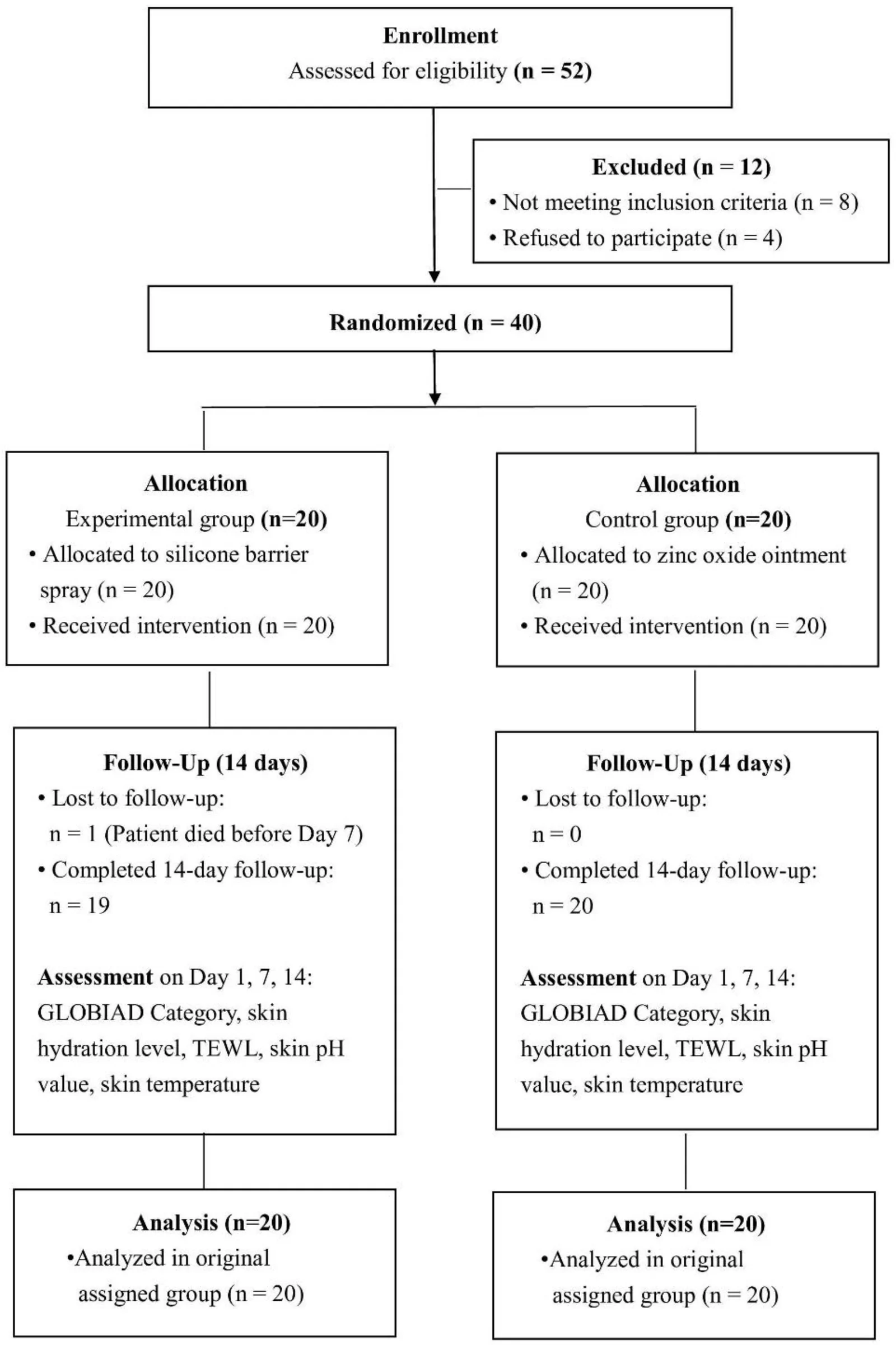
CONSORT flow diagram of participant screening, randomization, allocation, follow-up, and analysis. In accordance with the intent-to-treat principle, all 40 randomized participants (n = 20, experimental group; n = 20, control group) were included in the final analysis using generalized estimating equations (GEE) to accommodate the single missing follow-up dataset resulting from one participant's death before Day 7 in the experimental group.

**Figure 2:**
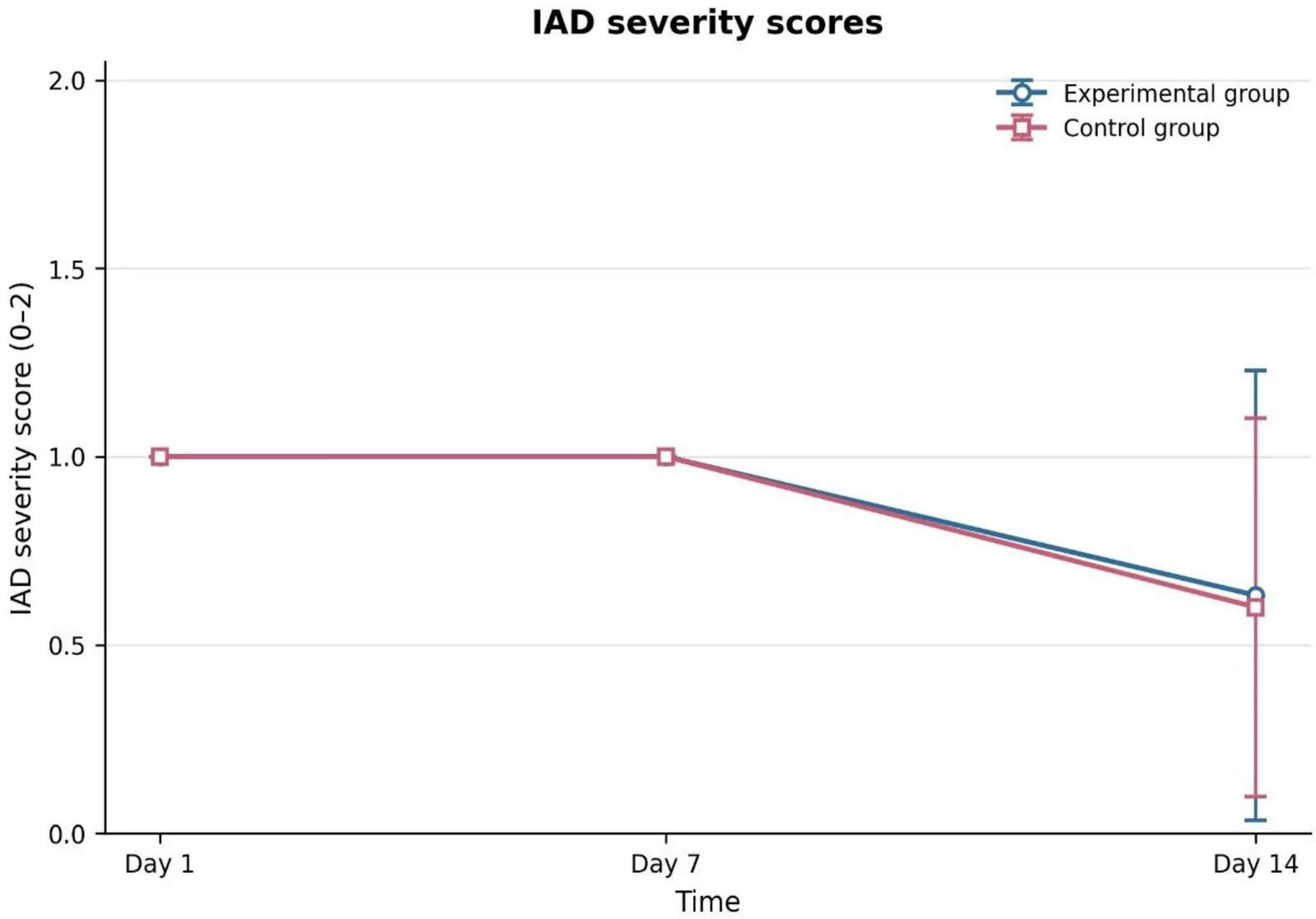
Change in incontinence-associated dermatitis (IAD) severity scores (0-2 scale) over time in the experimental and control groups. Values are presented as the mean ± standard deviation (SD). IAD severity is assessed using the GLOBIAD-M tool (0 = normal skin, 1 = GLOBIAD 1A, 2 = GLOBIAD 1B).

**Figure 3:**
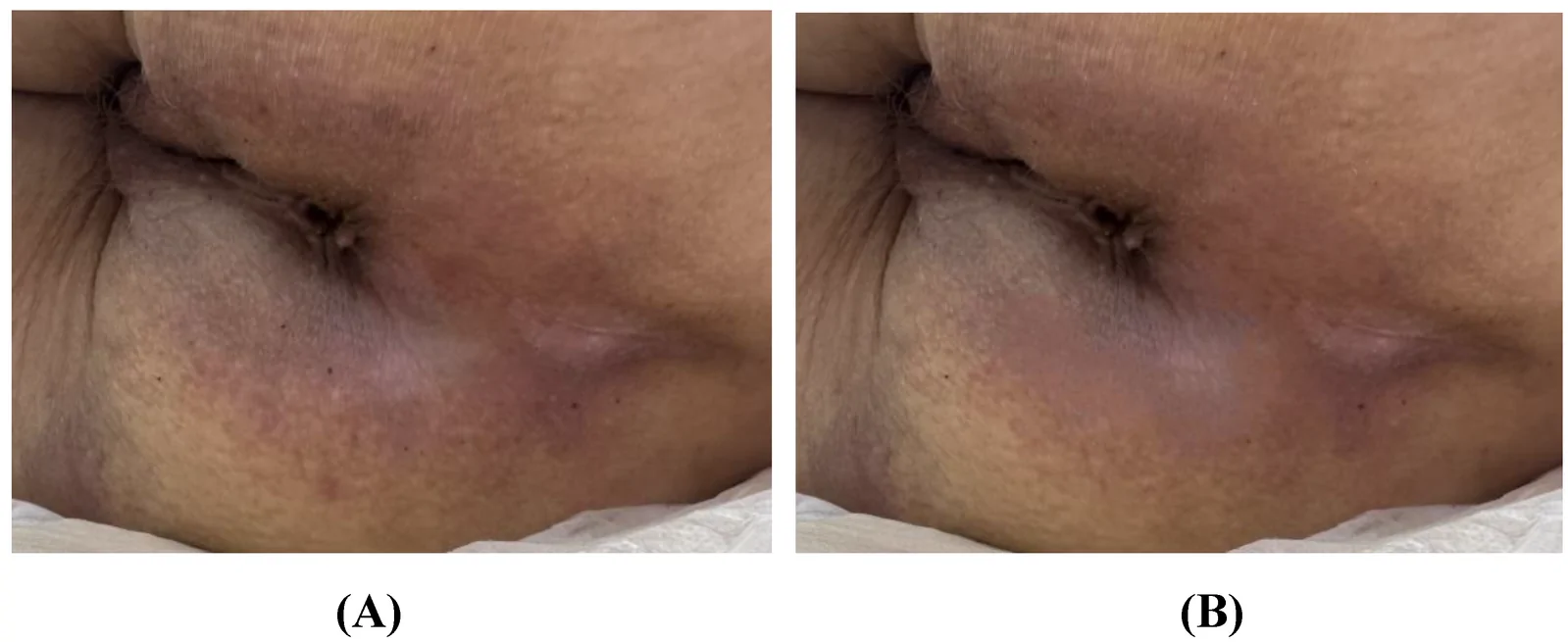
Representative clinical photographs of incontinence-associated dermatitis (IAD) before and after treatment.** (A)** Baseline (Day 1), showing persistent erythema without clinical signs of infection, and classified as GLOBIAD Category 1A. **(B)** Day 14 after treatment with sprayable silicone barrier film, showing reduced erythema and marked improvement of the affected skin compared with baseline.

**Figure 4:**
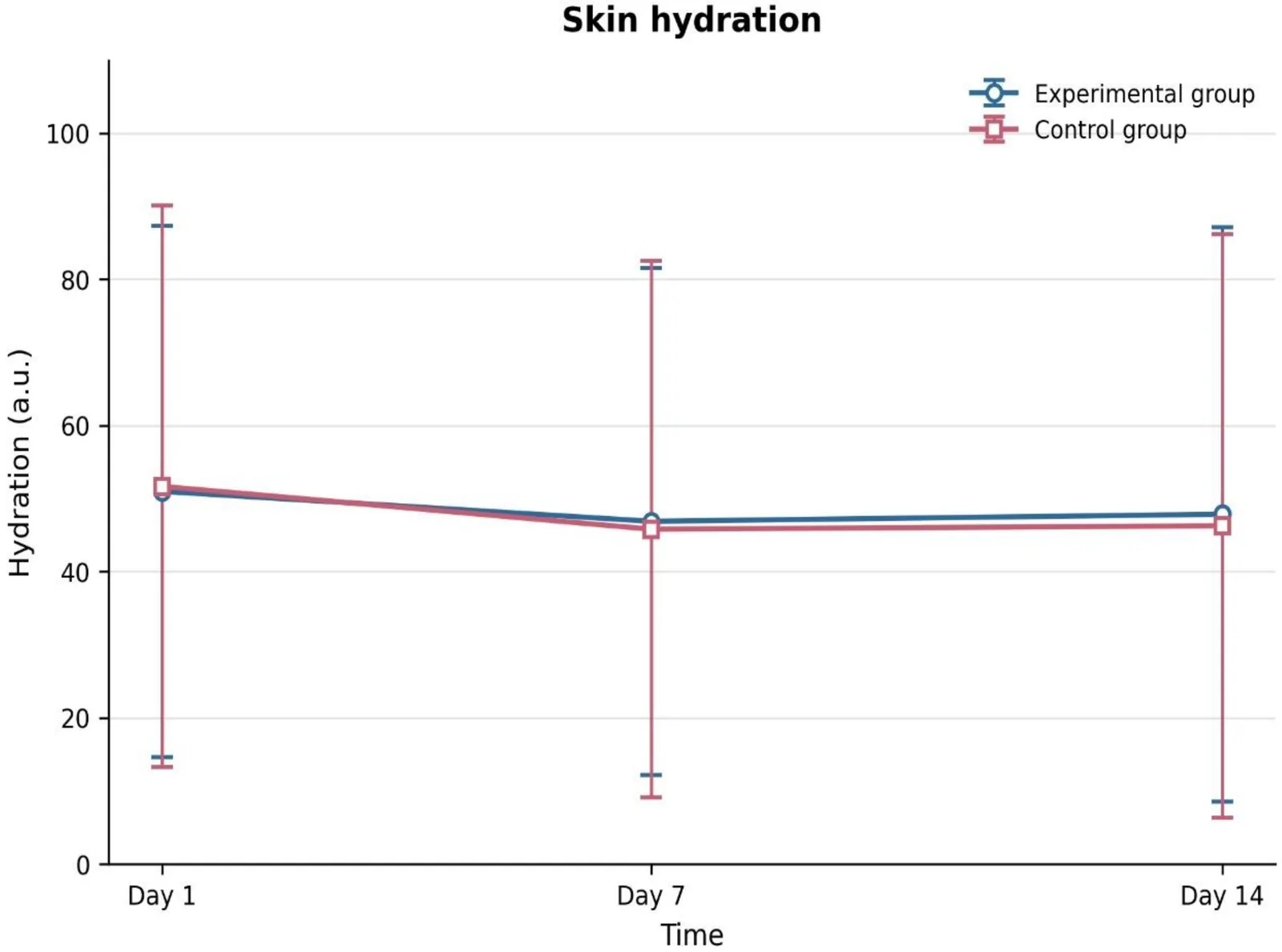
Skin hydration over time in the experimental and control groups. Values are presented as the mean ± standard deviation (SD).

**Figure 5:**
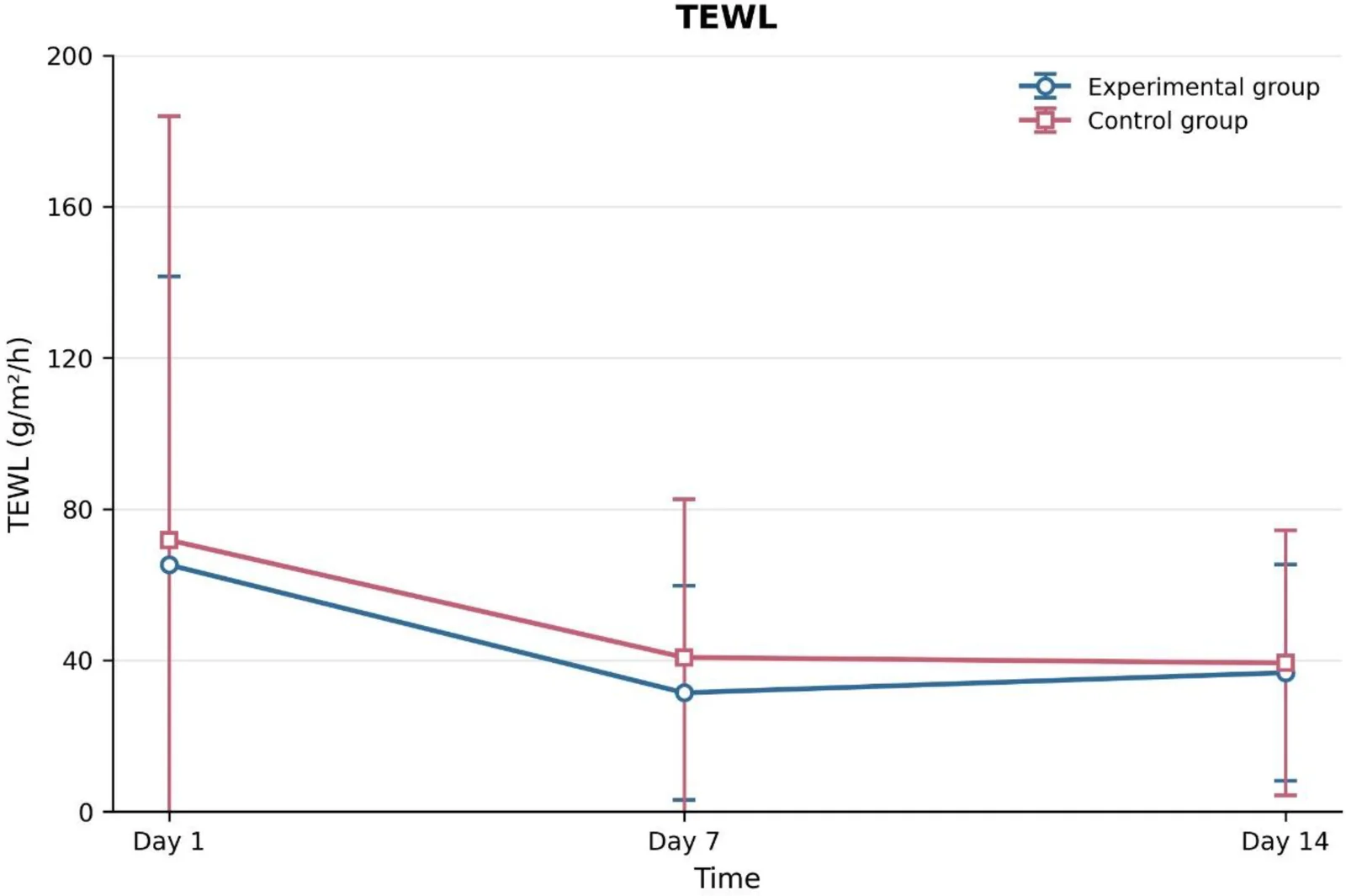
Transepidermal water loss (TEWL) over time in the experimental and control groups. Values are presented as the mean ± standard deviation (SD).

**Figure 6:**
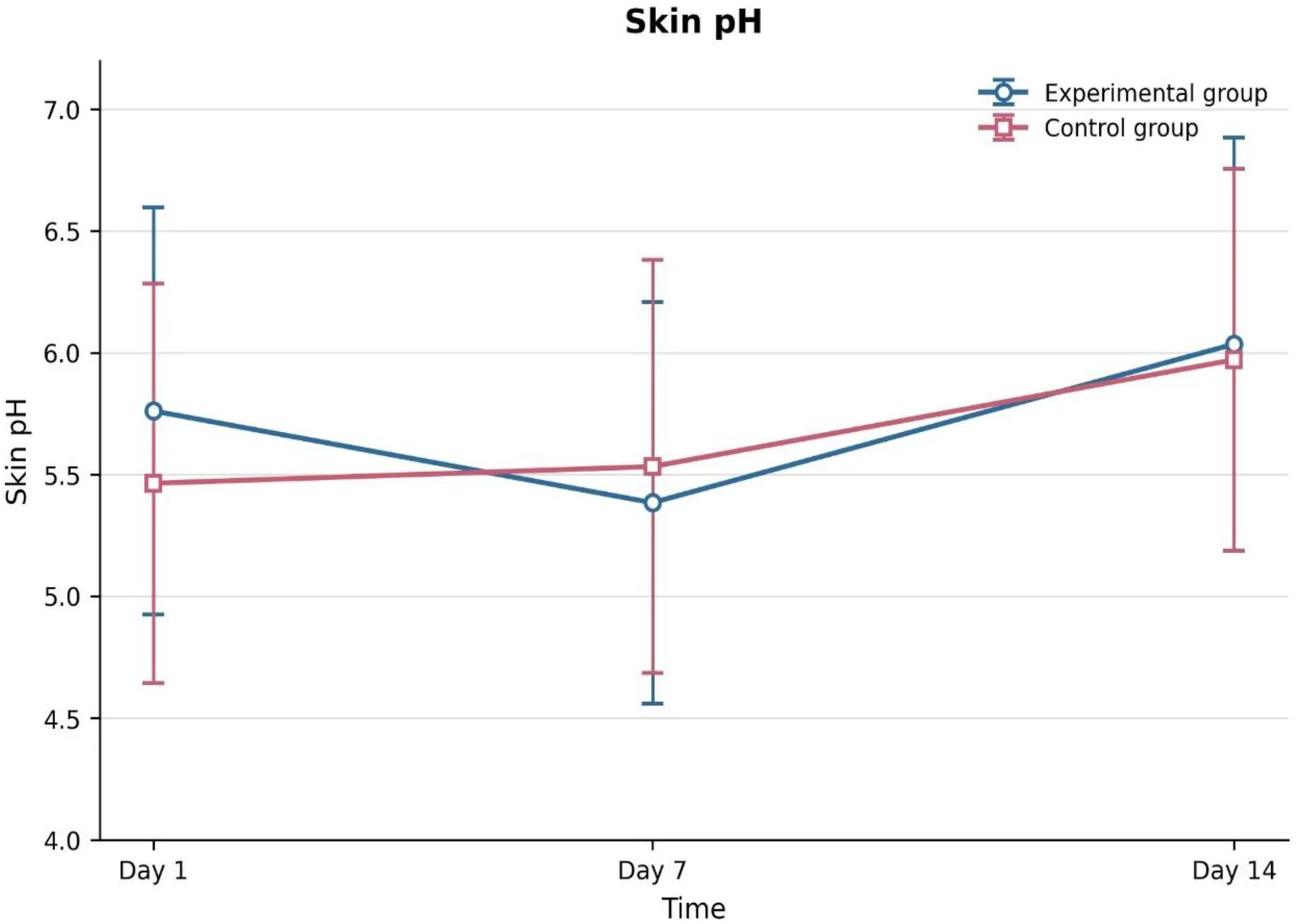
Skin pH over time in the experimental and control groups. Values are presented as the mean ± standard deviation (SD).

**Figure 7:**
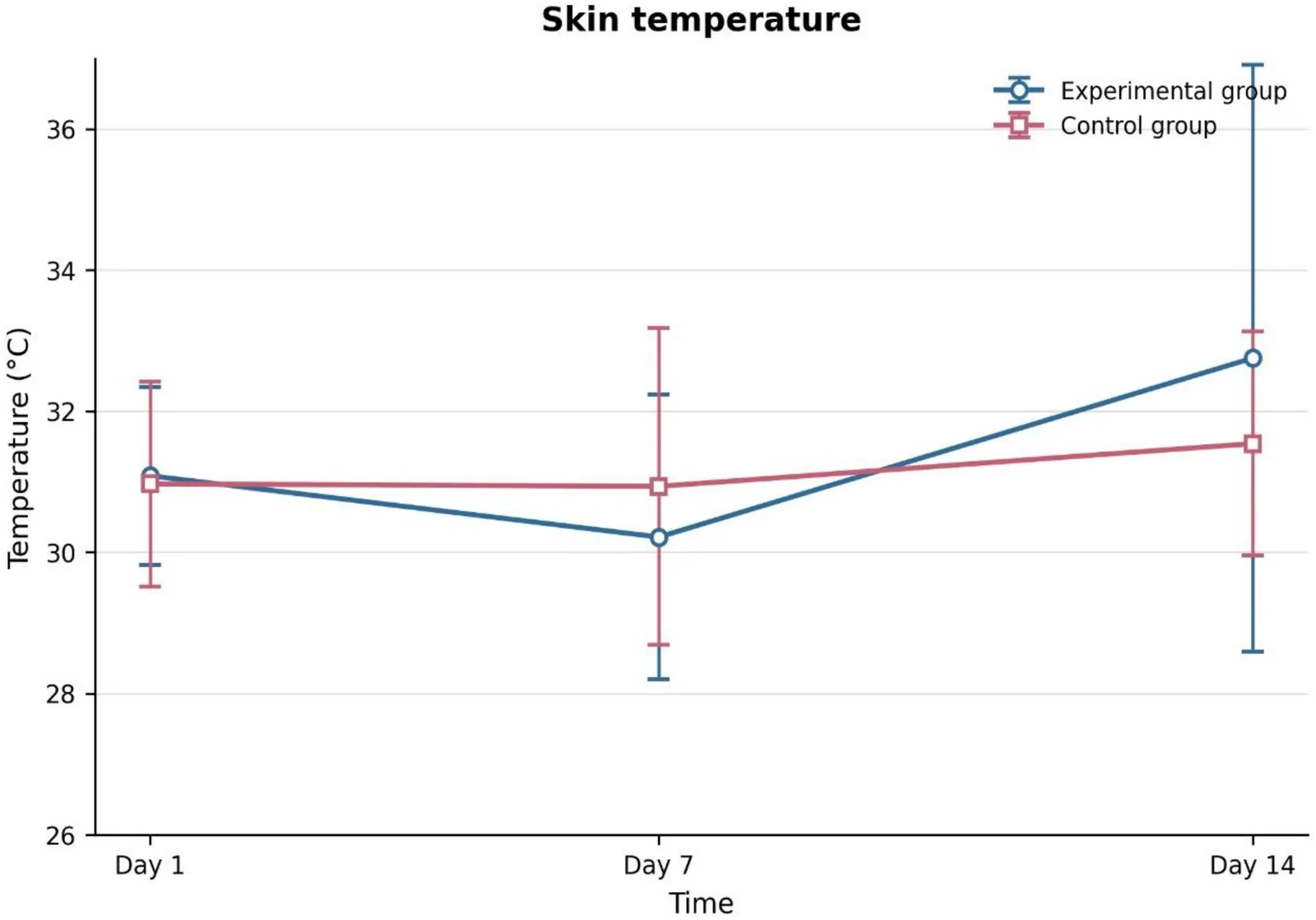
Skin temperature over time in the experimental and control groups. Values are presented as the mean ± standard deviation (SD).

**Figure 8:**
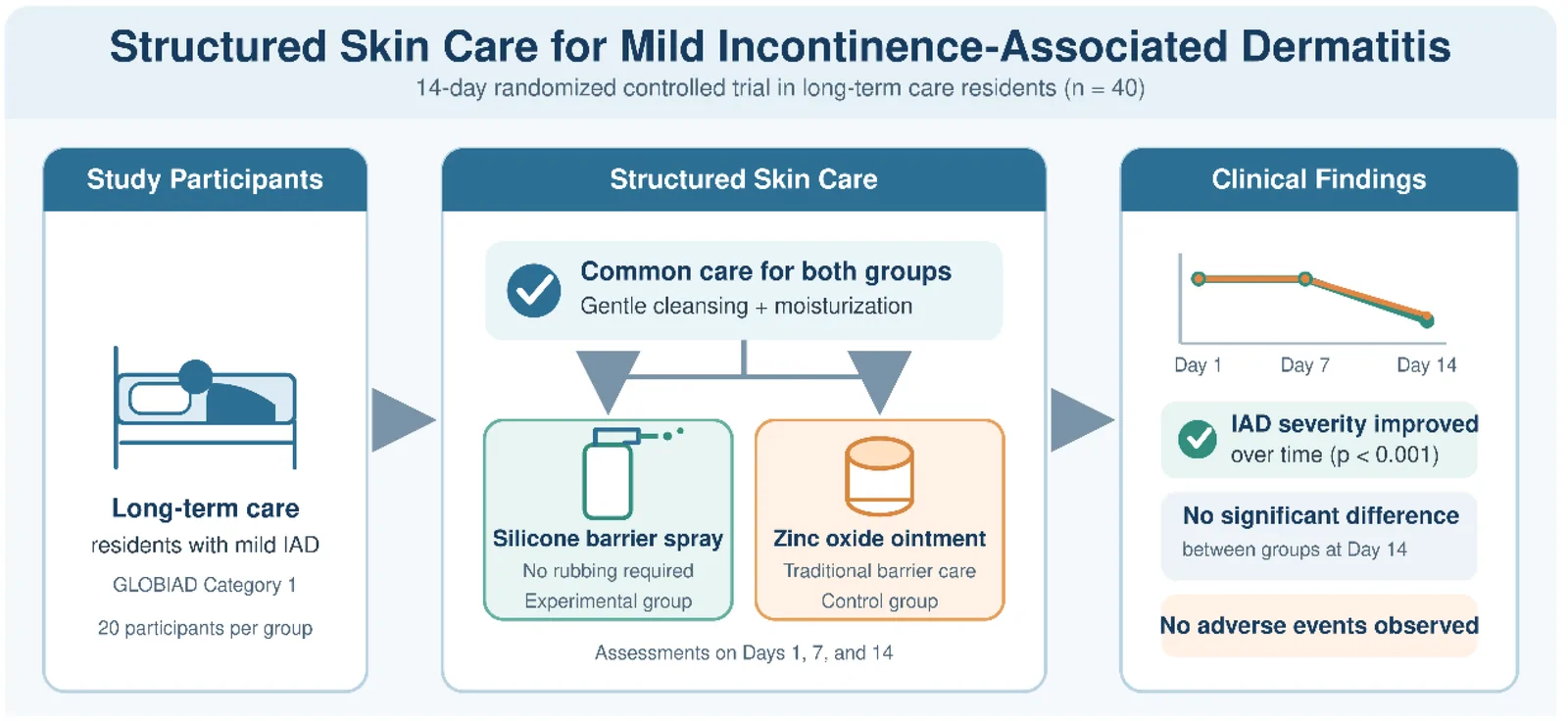
Schematic overview of structured skin care and main clinical findings in residents with mild incontinence-associated dermatitis (IAD). Both groups received gentle cleansing and moisturization, followed by barrier protection with either sprayable silicone barrier film or zinc oxide ointment. IAD severity improved significantly over time (p < 0.001). Neither the between-group difference at Day 14 nor the group × time interaction was statistically significant. No adverse events were observed.

**Table 1:** Baseline characteristics of participants.

Characteristic	Overall (n=40)	Experimental (n=20)	Control (n=20)	p-value
Age, years, mean ± SD	81.17 ± 10.46	81.25 ± 11.05	81.10 ± 10.12	0.965
Female, n (%)	28 (70.0)	13 (65.0)	15 (75.0)	0.490
BMI, mean ± SD	22.63 ± 3.07	22.54 ± 3.10	22.73 ± 3.12	0.850
Diabetes, n (%)	16 (40.0)	8 (40.0)	8 (40.0)	1.000
Malignancy, n (%)	2 (5.0)	0 (0.0)	2 (10.0)	0.147
Cardiovascular disease, n (%)	31 (77.5)	17 (85.0)	14 (70.0)	0.256
Pulmonary disease, n (%)	3 (7.5)	2 (10.0)	1 (5.0)	0.548
Dementia, n (%)	12 (30.0)	5 (25.0)	7 (35.0)	0.490
Stroke, n (%)	19 (47.5)	9 (45.0)	10 (50.0)	0.752

**Note:** Continuous variables are presented as the mean ± standard deviation (SD) and compared using Student's t-test. Categorical variables are presented as n (%) and compared using the chi-square test or Fisher's exact test, as appropriate.

**Table 2:** Baseline biophysical parameters.

Parameter	Experimental (n = 20)	Control (n = 20)	p-value
IAD severity score (0-2)	1.00 ± 0.00	1.00 ± 0.00	—
Hydration (a.u.)	50.99 ± 36.35	51.66 ± 38.44	0.955
TEWL (g/m²/h)	65.21 ± 76.21	71.78 ± 112.09	0.830
Skin pH	5.76 ± 0.84	5.46 ± 0.82	0.264
Skin temperature (°C)	31.09 ± 1.26	30.97 ± 1.45	0.791

**Note:** Values are presented as mean ± standard deviation (SD). IAD severity score is defined as 0 = normal skin, 1 = GLOBIAD 1A, 2 = GLOBIAD 1B. TEWL: transepidermal water loss.

**Table 3:** GEE analysis of longitudinal changes in IAD severity and biophysical parameters.

Outcome	Day 1	Day 7	Day 14	Day 14 between-group β (95% CI), *p*	Time effect *p*	Group × time *p*
IAD severity score (GLOBIAD-M)	1.00 ± 0.00	1.00 ± 0.00	0.62 ± 0.54	0.03 (-0.31 to 0.37), p = 0.855	<0.001	0.855
Hydration (a.u.)	51.32 ± 36.93	46.33 ± 35.28	47.05 ± 39.11	1.58 (-22.64 to 25.81), p = 0.898	0.734	0.985
TEWL (g/m²/h)	68.49 ± 94.67	36.14 ± 35.80	37.97 ± 31.65	-2.55 (-22.05 to 16.95), p = 0.798	0.320	0.885
Skin pH	5.61 ± 0.83	5.46 ± 0.83	6.00 ± 0.81	0.06 (-0.44 to 0.56), p = 0.801	0.036	0.355
Skin temperature (°C)	31.03 ± 1.34	30.58 ± 2.14	32.13 ± 3.14	1.21 (-0.73 to 3.16), p = 0.222	0.378	0.150

**Note:** Values are presented as mean ± SD. The Day 14 between-group coefficient (β) represents the estimated difference between the experimental and control groups. Overall time effects and group × time interaction were assessed using omnibus Wald tests in the GEE models. GEE: generalized estimating equation; TEWL: transepidermal water loss; CI: confidence interval.
